# Detecting neurodegenerative pathology in multiple sclerosis before irreversible brain tissue loss sets in

**DOI:** 10.1186/s40035-019-0178-4

**Published:** 2019-12-09

**Authors:** Jeroen Van Schependom, Kaat Guldolf, Marie Béatrice D’hooghe, Guy Nagels, Miguel D’haeseleer

**Affiliations:** 1 Neurology Department, Universitair Ziekenhuis Brussel; Center for Neurosciences, Vrije Universiteit Brussel, Laarbeeklaan 101, 1090 Brussel, Belgium; 2 Radiology Department Universitair Ziekenhuis Brussel, Brussels, Belgium; 3 Nationaal Multiple Sclerose Centrum, Melsbroek, Belgium

**Keywords:** Brain atrophy, magnetic resonance imaging, multiple sclerosis, neurodegeneration, neurofilaments

## Abstract

**Background:**

Multiple sclerosis (MS) is a complex chronic inflammatory and degenerative disorder of the central nervous system. Accelerated brain volume loss, or also termed atrophy, is currently emerging as a popular imaging marker of neurodegeneration in affected patients, but, unfortunately, can only be reliably interpreted at the time when irreversible tissue damage likely has already occurred. Timing of treatment decisions based on brain atrophy may therefore be viewed as suboptimal.

**Main body:**

This Narrative Review focuses on alternative techniques with the potential of detecting neurodegenerative events in the brain of subjects with MS prior to the atrophic stage. First, metabolic and molecular imaging provide the opportunity to identify early subcellular changes associated with energy dysfunction, which is an assumed core mechanism of axonal degeneration in MS. Second, cerebral hypoperfusion has been observed throughout the entire clinical spectrum of the disorder but it remains an open question whether this serves as an alternative marker of reduced metabolic activity, or exists as an independent contributing process, mediated by endothelin-1 hyperexpression. Third, both metabolic and perfusion alterations may lead to repercussions at the level of network performance and structural connectivity, respectively assessable by functional and diffusion tensor imaging. Fourth and finally, elevated body fluid levels of neurofilaments are gaining interest as a biochemical mirror of axonal damage in a wide range of neurological conditions, with early rises in patients with MS appearing to be predictive of future brain atrophy.

**Conclusions:**

Recent findings from the fields of advanced neuroradiology and neurochemistry provide the promising prospect of demonstrating degenerative brain pathology in patients with MS before atrophy has installed. Although the overall level of evidence on the presented topic is still preliminary, this Review may pave the way for further longitudinal and multimodal studies exploring the relationships between the abovementioned measures, possibly leading to novel insights in early disease mechanisms and therapeutic intervention strategies.

## Background

Multiple sclerosis (MS) is a chronic inflammatory demyelinating and degenerative disorder of the central nervous system (CNS) affecting over 2.5 million people worldwide. The first symptoms usually occur during young adulthood, after which patients may experience a heterogeneous relapsing or progressive disease course with a modulating role for age, sex and comorbidities [1–5]. Clinical outcomes are difficult to predict but cumulative axonal loss is generally seen as the primary determinant of long-term prognosis. Brain volume (BV) reduction, or atrophy, has recently emerged as a popular magnetic resonance imaging (MRI) marker of neurodegeneration in MS, pathologically corresponding with demyelination, decreased axonal count and neuronal cell death [6, 7]. In healthy individuals, the rate of BV decline, ranges between 0.2 and 0.5% per year, depending on the age of the subjects [8], whereas this process appears to advance approximately 3 to 5 times more rapid in individuals with MS. [9] Brain atrophy is present and clinically relevant in all subtypes, from subjects with a clinically isolated syndrome (CIS) to those with longstanding progressive disease [10, 11], being more pronounced in the later stages but seemingly proceeding at a similar pace throughout the entire clinical spectrum [12]. At the group level, development of brain atrophy, particularly in the grey matter, has become recognized as an independent predictor of current and future physical disability, mood disturbances and cognitive impairment; notably outperforming classical MRI markers of white matter inflammation [10, 13].

**Fig. 1:**
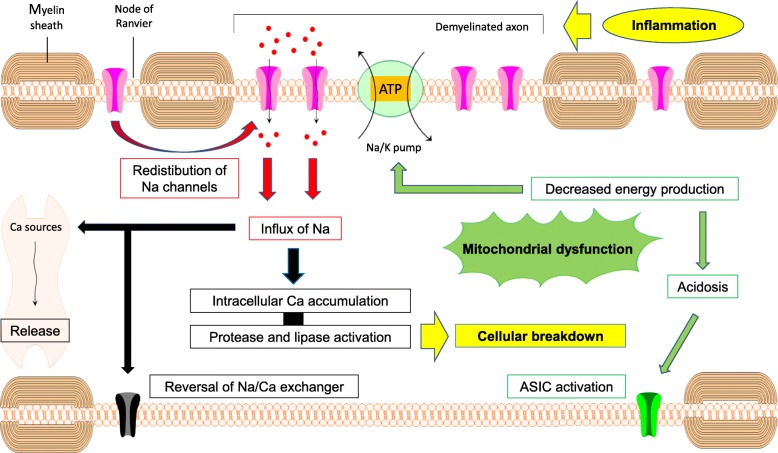
Cascade of events potentially linking inflammatory activity to slowly progressive neurodegeneration in MS. Chronic inflammatory demyelination leads to redistribution of sodium channels along the denudated axolemma, resulting in sodium influx. Elevated intracellular sodium levels increase the work-load of the energy-dependent Na/K pump. Mitochondrial function is impaired in multiple sclerosis (mainly resulting from the oxidative stress associated with inflammatory activity) which causes insufficient energy supply to compensate this imbalance. Intracellular sodium levels rise, leading to accumulation of calcium, e.g. by reversal of the transmembrane Na/Ca exchanger and release from intracellular sources. Acidosis contributes to sodium and calcium influx through opening of acid-sensing ion channels. Calcium stacking induces protease and lipase activity, eventually ending with cellular breakdown. ATP: adenosine triphosphate, ASIC: acid-sensing ion channel, MS: multiple sclerosis

The therapeutic landscape of MS is changing dramatically since the past 10 to 15 years [14], (Table 1) and the arrival of more potent drugs raised the standards of optimal disease control along this process. It has been suggested that achieving a ‘no evidence of disease activity’ (NEDA) status; defined as the absence of clinical relapses, new or enlarging MRI lesions and sustained progression on the Expanded Disability Status Scale (EDSS) [15]; (Figure 1) 2 years after starting disease-modifying treatment is predictive of a favourable outcome over up to 7 years [16]. This concept has subsequently found its way into clinical practice and research. Most first-line drugs have initially showed only a modest impact on brain atrophy but after the first year or treatment, when the possible confounding effects of pseudo-atrophy - i.e. the volume decrease resulting from oedema reduction in response to the anti-inflammatory therapy - can be eliminated, various drugs do seem to slow down the rate of BV loss [17–21]. Moreover, this effect looks to be more pronounced for second-line agents and correlates for a substantial degree with the treatment effect on disability [22]. Brain atrophy measures now start to become increasingly incorporated as supplementary endpoints in MS treatment trials, expanding the goal from NEDA-3 to NEDA-4 (the latter can be defined as the combined absence of clinical relapses, new/enlarging MRI lesions, sustained EDSS progression and accelerated rate of BV loss – a threshold of 0.4% decline per year has been suggested for the brain atrophy parameter) [23].

**Table 1:** Development timeline for disease-modifying therapy in MS

FDA or EMA approval^	Product	Main immunological mechanism of action
1993/1996	INF β-1b/-1a	Induces changes in cytokine balance
1996	Glatiramere acetate	Interferes with antigen presentation to T-lymphocytes
2000	Mitoxantrone	Reduces the number of circulating leukocytes
2004	Natalizumab	Blocks leukocyte migration across the BBB
2010	Fingolimod	Prevents lymphocyte egression from the lymph nodes
2012	Teriflunomide	Inhibits lymphocyte proliferation
2013	Dimethyl fumarate	Induces changes in cytokine balance
2014	Alemtuzumab Pegylated INF β-1a	Destructs circulating lymphocytes, followed by repopulation Induces changes in cytokine balance
2016°	Daclizumab	Induces immune tolerance
2017	Cladribine* Ocrelizumab	Reduces the number of circulating lymphocytes Depletes B-lymphocyte population
2019	Siponimod	Prevents lymphocyte egression from the lymph nodes
Pipeline	Ozanimod and ponesimod Ofatumumab Evobrutinib AHSCT	Prevents lymphocyte egression from the lymph nodes Depletes circulating B-lymphocytes Inhibits B-lymphocyte signaling and maturation Immune system reconstitution

*FDA* Food and Drug Administration, *EMA* European Medical Agency, *INF* interferon, *BBB* blood-brain barrier, *AHSCT* autologous stem cell transplantation, *MS* multiple sclerosis.

^ based on which approval came first

* No FDA approval at present

° Withdrawn in 2018

On the other hand, it must be stated that the implementation of brain atrophy into clinical MS practice develops more difficult than originally expected, mainly due to several unresolved biological and technical issues [10], and its use as a marker of disease progression is still not recommended according to academic imaging guidelines [24, 25]. An additional and even more important problem might be the reasonable likelihood that a substantial degree of irreversible tissue damage is already present at the time when brain atrophy becomes apparent. Expert opinion suggests that BV changes should be interpreted over a period of at least 2 years in the individual patient trajectory [8], and none of the currently available MS drugs have so far been able to reverse this phenomenon. Consequently, even under optimal methodological circumstances, real-life treatment decisions based on the detection of accelerated BV loss risk to fall well beyond the window of opportunity. This Review focuses on the question which alternative techniques might be able to demonstrate early signs of neurodegenerative brain pathology in subjects with MS, in the absence of atrophic changes and hopefully before structural tissue damage has become definite.

## Main text

### Molecular and metabolic imaging

#### Linking inflammation to neurodegeneration

We believe that the most logical approach to our postulated question would be an attempt to outline the biological processes taking place inside degenerating neurons of individuals with MS, at the stage where the cellular homeostasis becomes threatened, yet still preceding the imminence of cell death. The precise causative mechanism remains unknown but in the traditional teaching MS is initiated by auto-reactive lymphocytes entering the CNS from the peripheral immune system to provoke hallmark demyelinating lesions, also termed plaques [26, 27]. Axonal transection can occur during the formation of these plaques and subsequently trigger anterograde (Wallerian) and retrograde degeneration [28, 29]. In general, however, most axons will survive such acute inflammatory activity [30], and various radiological and neuropathological studies have demonstrated only a modest correlation between total lesion burden and axonal loss [31–35]. Acute tissue damage due to inflammation therefore does not seem to be a major contributor to brain atrophy in MS. Nevertheless, longitudinal MRI observations have revealed a more slowly proceeding axonal decay within chronic inactive plaques [36], and post-mortem studies in highly disabled subjects with MS have reported a reduction in the corticospinal axonal count of up to 70% [37]. Sustained demyelination is believed to be the responsible element, by triggering a cascade of events that will eventually, when initial compensatory mechanisms have become exhausted, evolve into axonal degeneration. Prominent factors associated with this pathway include oxidative stress, mitochondrial injury, energy failure and ionic dysregulation [30].

Chronic demyelination leads to loss of trophic support and redistribution of sodium channels along the denuded axolemma which, under normal physiologic circumstances, would have remained concentrated at the nodes of Ranvier. Extracellular sodium is expected to migrate inwards through these channels, based on the transmembrane concentration difference, implying that the energy-consuming Na/K pump will have to work at an increased rate to maintain correct ionic gradients and polarization across the cell membrane [38–40]. Axons highly depend on mitochondrial activity for local energy production, whereas it is precisely mitochondrial integrity that can become compromised in subjects with MS. [41, 42] Inflammatory responses are associated with the production of ROS and NO by activated cells of the innate immune system (i.e. microglia and macrophages), and mitochondria appear to be extremely vulnerable to this kind of oxidative stress. For instance, significant reductions of respiratory chain cytochrome c-oxidase I and complex IV enzymes in active MS lesions have been attributed to the effects of ROS and NO. [30, 43, 44] As a consequence, demyelinated axons may experience a mismatch between higher energy needs and decreased mitochondrial metabolic functioning. The increased mitochondrial density and activity, as seen in chronically inactive plaques [45, 46], likely act as initial compensatory adaptations, but one can assume that an overt energy deficiency arises once the oxidative damage surpasses a certain threshold. The Na/K pump may start to slow down when energy supply is no longer sufficient, resulting in intracellular sodium accumulation which, in turn, can lead to a rise of intracellular calcium, due to reversal of the transmembrane Na/Ca exchanger and release from intracellular calcium sources such as the endoplasmic reticulum. Exorbitant intracellular calcium release is recognized as a common final pathway of cellular death, as calcium can activate various lipases and proteases (e.g. ubiquitin and calpain), which cause breakdown of structural components and the process hereby to reach a point-of-no-return [30, 43, 44]. In addition, a number of self-reinforcing mechanisms are hidden within this cascade. Energy dysfunction will, for instance, lead to intracellular acidosis, promoting both supplementary sodium and calcium entrance by activation of acid-sensing ion channel (ASIC) 1a [43, 47]. Intracellular calcium stacking can support further sodium influx by opening other types of ion channels in the cell membrane [43], and the whole process of mitochondrial and diffuse structural damage will generate even more oxidative stress, ultimately ending in a detrimental vicious circle (overview presented in Figure 1).

Molecular and metabolic imaging provide the exciting ability to reflect some of these proposed subcellular alterations throughout the otherwise normal-appearing white and grey matter of patients with MS. Molecular imaging compromises advanced MRI applications in which advanced contrast agents are used to label the biological target, and positron emission tomography (PET) which relies on radioligands selectively binding to the proteins of interest. Metabolic imaging techniques, in turn, exploit the magnetic properties of atomic nuclei, such as protons or sodium ions. The idea that early neurodegenerative MS pathology can be captured in the absence of atrophy is thus far best supported by magnetic resonance spectroscopy (MRS), sodium imaging, chemical exchange saturation transfer (CEST) and PET findings.

#### Mitochondrial failure

N-acetyl-aspartate (NAA) is almost exclusively produced by neuronal mitochondria in mature CNS and figures as a marker of mitochondrial function and/or integrity in both white and grey matter [48, 49]. The precise biological functions remain unknown, but during early postnatal development NAA is transported in large quantities to oligodendrocytes and subsequently metabolized into building blocks for myelin formation [49]. Levels in the brain and spinal cord can be measured with ^1^H-MRS and are usually expressed as NAA/creatine ratios for reasons of stability [43, 50, 51]. Because mitochondrial failure appears to be a crucial feature of neurodegeneration in patients with MS, regional estimates may provide valuable insights on local neuronal health. In acute MS lesions, NAA is decreased when compared to surrounding normal-appearing white matter and the white matter of controls subjects [52, 53]. In part this may still be explained by inflammatory oedema, but an analogue reduction has also been observed in chronic white matter plaques appearing hypointense on T1-weighted MRI [54]. MRS studies of both relapsing and progressive patients with MS have fairly consistently demonstrated reduced concentrations in the normal-appearing white and grey matter, as compared to controls, with a tendency of being more pronounced in progressive stages and related to clinical disability [54–57].

Definite proof that a chronic NAA reduction unequivocally precedes brain atrophy in a longitudinal neurodegenerative scenario affecting patients with MS, and which is detectable within this context by MRS, is still lacking, but at least a few interesting findings can be extracted from the current literature.

First, MRS follow-up studies of acute lesions in the brain and spinal cord have demonstrated that reduced NAA, albeit not necessarily a return to normal, partially recovered over time [52, 58, 59]. Interestingly, progressive atrophy occurred at the same time as the NAA restoration (the latter likely reflecting an adaptive change of the surviving mitochondria), thus creating a time line in which the NAA decrease did precede atrophy, and where a partial causal relation between both may not be excluded. Kirov and co-workers found that NAA concentrations in cerebral normal-appearing white matter of recently diagnosed and mildly disabled individuals with MS, which were all on disease-modifying treatment, were 6% lower than in control subjects, but significantly increased over the next 3 years [60]. These studies support the concept that reduced NAA levels may be reversible and subject to therapeutic intervention (at least when they reflect metabolic alterations), whereas this has not yet been the case with atrophy. Second, reduced levels of NAA have also been reported in the brain of patients with a clinically and even radiologically isolated syndrome (RIS) [61–63], suggesting that mitochondrial dysfunction may already occur at the earliest stages of the disease, where less atrophy would be expected. In contrast, one study found an isolated decrease in normalized global brain and cortical volumes of subjects with a RIS. However, a trend in NAA decrease was still observed in those which were at high risk for later MS conversion [64]. Third, lower NAA concentrations were measured in the spinal cord of 21 early primary progressive MS patients without extensive cord atrophy, and were associated with higher EDSS scores [65]. Fourth, MRS is also able to quantify cerebral glutamate, an excitatory neurotransmitter, which has been found to be increased in the normal-appearing white matter of all subtypes of MS, when compared with healthy controls [66, 67], potentially due to increased production by inflammatory cells in combination with reduced clearance by oligodendrocytes and astrocytes [48]. Later on, however, there are indications of a continuous glutamate decline [67], in contrast to NAA levels which remain relatively stable as a likely result of numeric mitochondrial adaptation. Glutamate itself may act as an inductor of neurodegeneration, by influencing transmembrane sodium and calcium channels in a mechanism termed excitotoxicity [68]. Higher concentrations in the normal-appearing white matter of a large cohort predominantly consisting of patients with a relapsing-remitting subtype, were associated with a more pronounced NAA decline (after 2 years) and BV reduction (after 3 years) over time [66]. When glutamate levels are normalized for NAA, they will probably provide more accurate information on its assumed neurodegenerative stimulus, because this ratio corrects for the amount of already existing neuronal loss. Longitudinal studies exploring the temporal relationship between glutamate/NAA, NAA/creatine and BV within this context would be very much of interest.

#### Sodium accumulation

Intraneuronal sodium accumulation is an early transduction of energy dysfunction in our proposed cascade of neurodegenerative events in MS. Under normal physiological circumstances, CNS sodium concentrations are actively kept low in the intracellular compartment (tissue fraction 80%), as compared to those in the smaller extracellular counterpart (20%) [48], in order to preserve a correct transmembrane electric gradient. In vivo, sodium can be quantified using ^23^Na MRI, based on which several groups have found increased concentrations within plaques, normal-appearing white matter and cortical and deep grey matter of subjects with MS, as compared to controls [69–71]. Higher values appear to be linked with progressive disease and greater disability [70]. In a combined ^23^Na MRI and MRS analysis, sodium accumulation was directly associated with neuronal metabolic dysfunction, as reflected by decreased NAA levels, in focal lesions, grey matter and normal-appearing white matter of 21 individuals with relapsing-remitting MS. [72]

The abovementioned studies are somewhat limited by the fact that they have assessed total sodium concentrations without distinguishing between the intra- and extracellular fraction, and theoretically, it cannot be excluded that an augmentation of total levels is predominantly driven by expansion of the extracellular space, in parallel with neuronal atrophy. However, abnormally high sodium concentrations were also found in grey matter zones which were spared by atrophy in progressive MS [73], as well as in isolated brain regions of patients with an early relapsing-remitting course [71], suggesting that sodium accumulation can be a marker of neuronal metabolic dysfunction rather than merely resulting from BV loss. More compelling evidence for this theory was provided by Petracca and co-workers who performed an ultra-high field (7 T) triple-quantum filtered ^23^Na MRI study enabling the quantification of the intracellular sodium concentration and extracellular sodium fraction, an indirect measure of the extracellular sodium concentration. They reported an increase of both total and intracellular sodium levels in the grey and normal-appearing white matter of 19 patients with relapsing-remitting MS, as compared to healthy controls, whereas extracellular sodium fractions in fact were reduced, arguing against the simultaneous presence of atrophy [74]. Interestingly, in a recent French publication cognitive impairment in relatively early relapsing-remitting MS was better explained by total sodium accumulation then volume loss in the grey matter [75], while a British group reported that increased total cortical levels were associated with multiple physical and cognitive outcome measures (e.g. EDSS and Symbol Digit Modalities Test scores) in patients with a relapsing onset, independent of grey matter atrophy [76].

#### Intracellular acidosis

CEST allows to image compounds at concentrations that are too low to be detected using standard MRI. The technique is based on the exchange of protons between specific tissues, that are first saturated, and water [77]. One of the most common applications is the assessment of amide proton transfer (APT) effects in proteins and peptides, which has been successful at demonstrating elevated protein concentrations in brain tumours, as well as at the detection of acute ischemic stroke, the latter due to the sensitivity of the APT effect to pH changes [78]. Within the field of MS, a 7 T study - including only 4 subjects - delivered preliminary evidence that the APT signal varied with lesion type and differed between plaques and healthy white matter [79]. A more recent study showed APT changes in the normal-appearing white matter of the cervical spinal cord of individuals with MS, as compared to controls, at 3 T [80]. Both acute inflammatory damage and chronic energy failure may result in intracellular acidosis, potentially accounting for the encountered alterations in these patients. Extreme or prolonged acidosis is toxic to CNS neurons in a process believed to be mediated by ASIC variants, and the positive effect of amiloride, which is an ASIC antagonist, on brain atrophy in subjects with primary progressive MS hints towards neuroprotective properties of blocking this pathway [81]. Although APT-CEST is a promising technique, its low specificity and dependence on acquisition parameters, manufacturers and coils, does currently not support a widespread clinical application. The lack of studies incorporating APT-CEST obstructs us to elaborate yet on whether this method is able to detect degenerative changes related to MS earlier than conventional imaging.

#### Positron emission tomography findings

MS is multifactorial disorder in which many different radioligands can be used for experimental PET imaging purposes, depending on whether the biological process of interest is inflammation, myelin breakdown, neuronal degeneration or astrocyte activation [48]. One of the best-known applications in human brain is fluorodeoxyglucose (FDG)-PET, which measures the rate of cerebral glucose consumption. Higher uptake, as compared to healthy controls, has been observed in patients with MS and was attributed to increased regional glucose utilization by activated inflammatory cells [82, 83], but it seems equally plausible for the global signal to become suppressed due to loss of neuronal metabolic integrity. In support of this hypothesis, FDG uptake was decreased in the spinal cord of 8 patients with MS, negatively correlating with autonomic and motor symptoms [84]. Based on FDG-PET imaging alone, however, the ability to discriminate structurally still uncomplicated metabolic dysfunction from overt atrophy in the brain seems rather unlikely.

^11^C-flumazenil is a different PET radioligand with selectivity for the benzodiazepine site of the class A gamma-aminobutyric acid (GABA) receptor complex in synapses of grey matter neurons. Freeman and co-workers found a reduced cortical ^11^C-flumazenil binding in a mixture of patients with relapsing-remitting and secondary progressive MS, as compared to healthy controls. Interestingly, this effect was already noticeable in the subset of relapsing-remitting subjects, who did not have a significant degree of grey matter atrophy, and was globally associated with reduced information processing speed [85]. Cao and co-workers described in a classical MRS study that GABA levels themselves were decreased in the posterior cingulate cortex and left hippocampus of patients with relapsing-remitting disease, which was correlated impaired verbal memory and executive function [86]. If and how dysfunctional GABA-minergic neurotransmission relates to neuronal metabolic dysfunction in MS remains unknown, but these findings do suggest that it can be detectable at a clinically relevant stage, in the absence of cortical volume loss.

Microglia are local macrophage residents that, together with infiltrating blood-derived monocytic cells, play a key role in regulating CNS inflammation. In MS, microglial activation may contribute to both tissue damage, mainly through the production of oxidative agents [87–89], and repair mechanisms, e.g. by scavenging for cell debris. Activation of microglia has been associated with increased PET signals in subjects with MS, as compared to heathy controls. The most investigated target is the translocator protein receptor (TSPO), which is normally expressed in small amounts by the outer mitochondrial membrane. TSPO studies using ^11^C-PK11195 have demonstrated increased uptake in active lesions, as well as in normal-appearing white and cortical grey matter, correlating with disability and brain atrophy measures [90–93]. Second generation TSPO tracers, such as ^11^C-PBR28, have been developed to improve affinity and specificity of the receptor binding and, interestingly, microglial activation was associated with brain volume loss at the cross-sectional and longitudinal level (correlation coefficients were -0.54 and 0.86, respectively) in two recent studies using this compound [94, 95].

### Cerebral blood flow

#### Cerebral hypoperfusion in multiple sclerosis

**Fig. 2:**
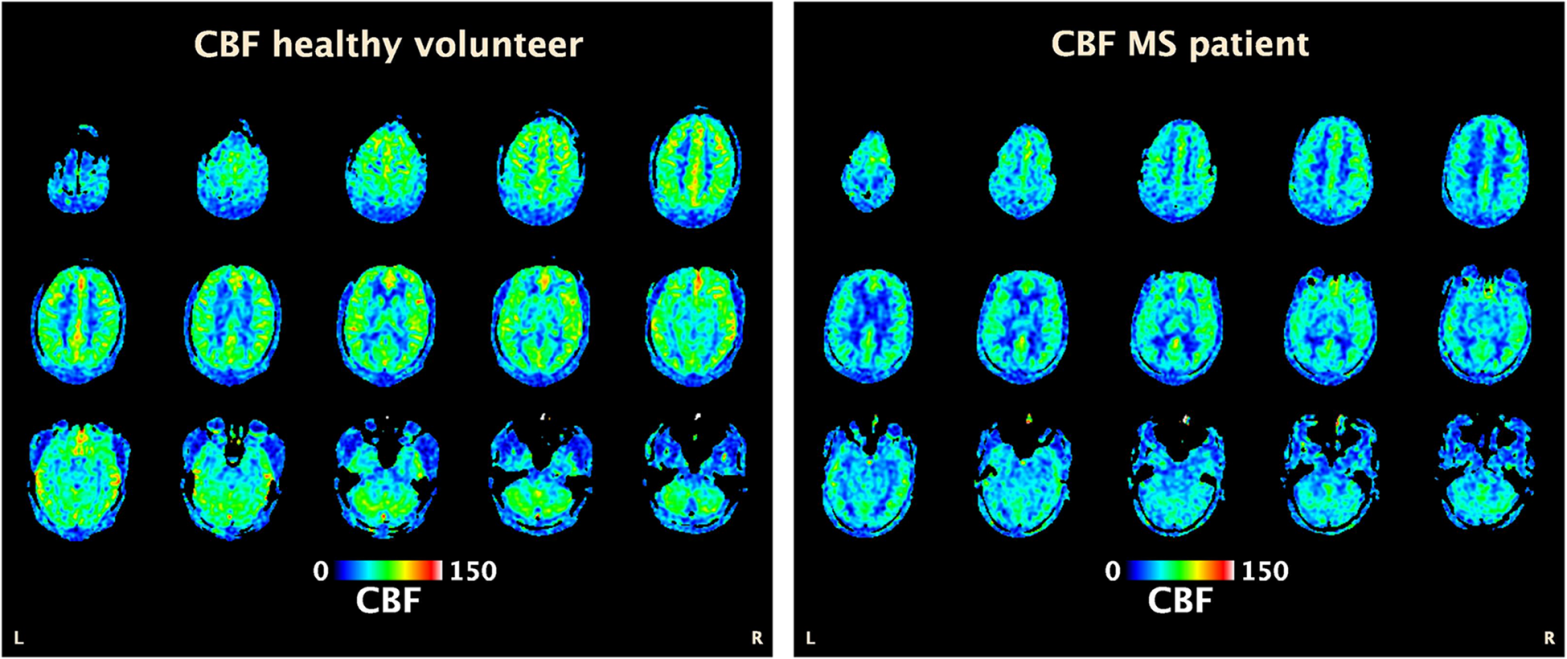
Brain perfusion is globally decreased in MS. Perfusion-weighted brain MRI (arterial spin labelling) from a 35-year old healthy male volunteer (left) versus a 34-year old man with relapsing-remitting MS (right), with CBF color-coded map overlay. Brain perfusion is globally reduced in the MS patients, as compared to the healthy volunteer. CBF: cerebral blood flow (expressed as mL/100 g/min), MS: multiple sclerosis, MRI: magnetic resonance imaging

Cerebral blood flow (CBF) is the most commonly used numerical expression of cerebral perfusion and represents the volume of blood flowing through a given amount of brain tissue per time unit [96]. As blood supply in the CNS is actively regulated by astrocytes to match regional metabolic activity [97], energy failure in MS would be expected to be accompanied by a reduction in CBF, which, theoretically, may serve as an alternative marker of the early neurodegenerative process. The first reports of cerebral hypoperfusion in patients with MS were published more than 3 decades ago, based on single-photon emission computed tomography and PET findings [98–101]. These studies, though, received little attention at the time due to technical limitations, such as low spatial resolution, and lack of conceptual understanding. The development of more accurate perfusion-weighted MRI methods has revived interest in this field during the past 15 years. Studies using dynamic susceptibility contrast-enhanced MRI have demonstrated a globally decreased CBF, as compared to healthy controls, in the normal-appearing white matter of patients with relapsing-remitting and primary progressive MS, as well as in those with a CIS [102–104]. Similar alterations have been found in the deep grey matter of patients with a CIS and relapsing-remitting MS. [105] Subsequently, global brain hypoperfusion, including in the cerebral cortex and cerebellum, was observed in mixed cohorts of MS subjects, by means of arterial spin labelling (ASL), a non-invasive technique which has the advantage of not requiring contrast administration [106, 107]; (see Figure 2 for an example from the authors’ own records, unpublished data). Overall, these findings suggest that reduced CBF is an early and integral feature of the disease, independent of its clinical course.

The exact underlying mechanism and pathophysiological significance of reduced brain perfusion in MS remain poorly understood at present. Somewhat surprisingly, Saindane and co-workers concluded that perfusion and diffusion tensor MRI properties of the normal-appearing corpus callosum were compatible with primary ischemia in relapsing-remitting patients, rather than with hypoperfusion secondary to impaired axonal integrity [108]. This theory was later supported by a combined ASL perfusion and MRS study, in which both CBF and NAA levels were lower in the centrum semiovale of individuals with MS, as compared to control subjects. NAA/CBF ratios in the MS group were significantly higher than in the controls, suggesting that the reduced perfusion is greater than what would be expected from decreased axonal metabolism (or loss) alone [51]. These findings might be explained by overexpression of the vasospastic agent endothelin-1 by reactive astrocytes in focal lesions, possibly induced through inflammatory cytokines, and subsequent release in the cerebral circulation [107, 109]. In support of this theory, microcirculatory disturbances, characterized by impaired arteriolar vasoreactivity measured with hypercapnic perfusion MRI, have recently been associated with MS. [110, 111]

#### Active involvement in disease pathology?

The idea that brain hypoperfusion precedes or even induces neurodegenerative changes in MS, is currently still speculative yet intriguing. Some pathological data do support a theoretical framework for this type of ‘reverse causality’ between both. Enhanced expression of hypoxia-inducible factor (HIF)-1α and its downstream genes, a universal salvage pathway that becomes activated in response to tissue hypoxia, has been observed in post-mortem normal-appearing white matter of patients with MS. [112] Cerebral white matter myelin and axons tissue appear to be particularly susceptible to chronic hypoxia [113], and animal models have demonstrated that chronically impaired brain perfusion induces mitochondrial dysfunction and production of free radicals - both of which are assumed to be involved in the progressive neurodegenerative process of MS - eventually resulting in axonal injury [114].

Prospective longitudinal imaging data are missing but several cross-sectional analyses have already suggested the presence of a functionally relevant hypoperfusion in non-atrophic brain regions of subjects with MS. Hence, Debernard and co-workers have found a global perfusion reduction in the cerebral cortex and deep grey matter of patients with early relapsing-remitting MS, compared to controls, in the absence of corresponding volume loss. This CBF reduction was positively correlated with objective memory disturbances [115]. A similar atrophy-independent perfusion decrease has been described in cortical and frontal grey matter of cognitively impaired versus non-impaired patients [116, 117]. Doche and co-workers evaluated grey matter CBF of subjects with relapsing-remitting MS and matched controls, in combination with voxel-based-morphometry analysis in order to exclude atrophic regions. Significant hypoperfusion was measured in both thalami of the MS subjects, and positively correlated with Multiple Sclerosis Functional Composite evaluations (i.e. combined assessment of mobility, dexterity and cognition), nearly reaching an additional significant negative correlation with EDSS scores [118]. Poor overlap between grey matter atrophy and CBF was, finally, also observed in a recent combined conventional 3D-T1 and ASL study [119].

### Connectivity assessment

#### Network structures

Some neurological functions are organized in a way that focal damage can cause very straightforward clinical impairment. For example, a single lesion in the middle temporal visual area, which is involved in the perception of motion, may lead to motion blindness [120]. Other functions, mainly concerning behaviour and cognition, appear to be distributed more diffusely within the brain, and rely on dynamic communication between regions operating in higher network structures. The pathological relevance of such network disruption is becoming increasingly recognized in various disorders, including MS, depression, Alzheimer’s disease and schizophrenia [121, 122]. Studies in these fields usually refer to 2 types of connectivity issues. The term structural connectivity embraces the direct anatomical linkage between different brain areas, while functional connectivity (FC) is based on the idea that remote regions expressing a similar temporal pattern of neuronal activation share information and are functionally connected [123]. One can assume that white matter tract integrity is an important prerequisite for correct interregional cross talk, whereas precisely myelin loss and axonal degeneration are hallmark features of MS pathology. In the hypothesis that energy failure, due to chronic demyelination and/or perfusion impairment, leads to early axonal dysfunction in subjects with MS, this might as well be reflected by changes at the network level.

#### Functional imaging

Functional MRI (fMRI) detects brain activity by measuring changes in CBF as a surrogate parameter, supported by the principle that regional perfusion and neuronal activation are coupled [97]. Blood oxygenation level dependent (BOLD) imaging is the standard technique for activity mapping in fMRI studies. Activation of brain tissue will lead to increased oxygen consumption in the neighbouring capillaries and venules, resulting in a decreased oxygenated/deoxygenated hemoglobin ratio. A compensatory augmentation in local CBF will take place over the next few seconds, delivering an immediate surplus of oxygenated hemoglobin. It is this pronounced rebound effect which will be visualized as the BOLD signal, because of the paramagnetic differences between both isoforms [124].

**Fig. 3:**
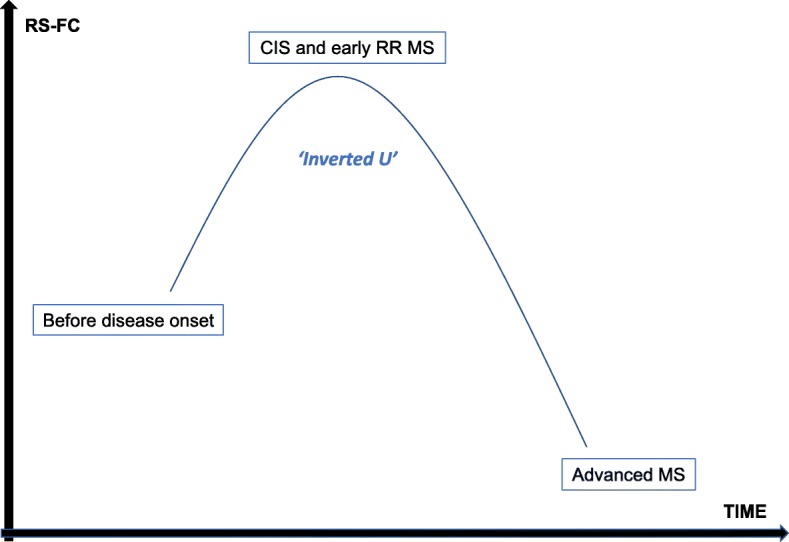
Longitudinal evolution of the default network’s RS-FC in MS. RS-CF is elevated early in the disease, as compared to healthy age-related controls, but subsequently drops down again, first to normal levels and then to reduced levels in more advanced patients. Impaired cognition seems to be associated with early increases and late decreases, most likely reflecting initial functional compensation followed by exhaustion of these mechanisms. RS-FC: resting-state functional connectivity, CIS: clinically isolated syndrome. RR: relapsing-remitting, MS: multiple sclerosis

The most primary application of fMRI explores BOLD responses during specific tasks or stimuli. Increased zones of activation while performing motor or cognitive exercises have been consistently demonstrated in patients with MS, as compared to healthy controls [125, 126]. These observations possibly reflect the capacity of the brain to recruit supplementary cortical areas to maintain a certain level of performance. Already in the CIS stage, patients showed increased activations and connectivity during the Paced Auditory Serial Addition Test [127]. In recent years, the analysis of the ‘brain-at-rest’ has emerged as an alternative to task-based studies, driven by advantages such as the ease and replicability of the paradigm, the fact that encountered differences are less likely to result from variations in task-performance, and the observation that large neuronal networks are activated during rest. Resting-state FC has been found to be substantially different in patients with MS, as compared to controls. In general, subjects in the CIS or early relapsing-remitting stage (disease duration less than 2 years) display an increased FC of the default network [128–130], i.e. the network that typically becomes activated during wakeful rest (i.e. conscious state but not engaged in a distinct mental activity) [131], with subsequent decrease to normal levels and reduced FC in more advanced patients [132–134], all compared to normal controls. Remarkably, impaired cognition hereby seemed to be associated with early elevations as well as late decrements of FC. These findings suggest an ‘inverted U-shaped’-like evolution of resting-state FC along the disease trajectory, ending in overt clinical deterioration when initial compensatory mechanisms have become overturned (Figure 3). Whether decreased axonal functioning prior to irreversible damage is sufficient to induce detectable network changes in MS is unknown, but this hypothesis may be supported by findings of disturbed network efficiency in CIS patients with reduced FC in occipital, temporal and frontal cortices [135].

#### Diffusion tensor imaging

**Fig. 4:**
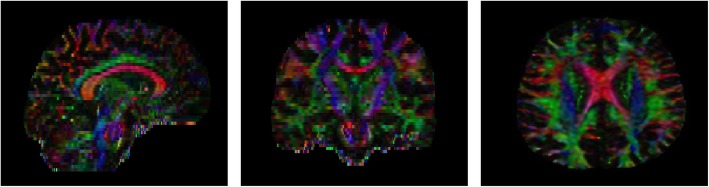
Examples of white matter tract orientation on processed DTI. Color denotes the direction of the first eigenvector at each voxel, weighted with the FA of that voxel. Red: left-right; blue: superior-inferior; green: anterior-posterior. Left: sagittal plane; middle: coronal plane; right: axial plane. DTI: diffusion tensor imaging, FA: fractional anisotropy

Diffusion tensor imaging (DTI) is a specific form of diffusion-weighted MRI that allows tracking of water molecules through biological tissues [136]. In a medium free from barriers, these molecules are expected to disperse equally in all directions (i.e. isotropic diffusion). Axons, however, are organized in parallel bundles and have surrounding myelin, resulting in an internal diffusion pattern which is not random but heavily favoured to follow the main directional axis of the fibres. Profiting from its relatively limited scan time duration, DTI is the most commonly used method to study structural connectivity in the human brain. Computer-based reconstructions can generate visual impressions called tractography, (Figure 4) and from a mathematical perspective, the diffusion tensor is generally summarized in 2 parameters: mean diffusivity (MD) representing the rate of random molecular diffusion (lower values correspond to low diffusivity), and fractional anisotropy (FA) as a normalized measure of the isotropic character of the diffusion (0 = isotropic; 1 = anisotropic). Although the interpretation of these scalar values depends on the brain region, cellular basis and the specific disease process [137], normal-appearing white matter of patients with MS typically shows an increase in MD and a decrease in FA [129, 138–141], pointing towards a reduced fibral integrity. Pre-atrophic disruption of axonal architecture, whether this involves myelin breakdown, axolemma leakage or both, could be reflected by similar DTI alterations.

In a 2 year follow-up study of 37 CIS patients, Rocca and colleagues observed a distributed pattern of increased white matter diffusivity at baseline (2-60 days after diagnosis), which remained stable for one year but resulted in a further increase at 24 months. In the same study, an early increase in grey matter volume of the bilateral middle and inferior temporal gyri was noted at baseline, followed by a further increase in several grey matter structures at 3 months and a volume decrease of the deep nuclei, cerebellar cortex and several cortical areas from month 12 onwards [142]. Whereas the early increase in grey matter volume may be explained by either pseudo-atrophy or structural plasticity (or a combination of both), the early increase in MD may either point to early microstructural changes or an effect similar to pseudo-atrophy. Indeed, inflammation induced swelling of the brain may allow water molecules to diffuse more freely, but the further increase in MD at 24 months could then reflect true axonal pathology. Yet, it is important to note that conflicting results have been reported in the literature: while the Rocca group observed an increase in MD without changes in FA, researchers at the Buffalo Neuroimaging Analysis Centre have reported reduced FA in different cortical and subcortical brain structures of 45 CIS patients without differences in MD [143]. Interestingly, DTI alterations have been found in the volumetrically normal hippocampus of CIS patients, as compared to healthy controls, and these were negatively correlated with episodic verbal memory performance, thus arguing that clinically relevant microstructural brain damage can be detected prior to atrophy [144].

The growing presence of DTI in MS research can additionally be illustrated by studies demonstrating pharmaceutical effects on diffusion parameters. In a recent longitudinal observation, a relatively stable pattern in diffusion metrics was encountered in patients with relapsing-remitting MS which were administered dimethyl fumarate (DMF). In the same study, healthy controls showed a significantly greater rate of diffusion parameters increase in the thalamus and normal-appearing white matter, compared to the subjects with MS [145], cautiously (due to small sample sizes and lack of a placebo-controlled arm) suggesting a supplementary neuroprotective effect of DMF. Other studies reported a significant FA increase and radial diffusivity decrease in the corticospinal tract after initiation of fingolimod [146], or a stable pattern of diffusion parameters in the normal-appearing white matter for up to 48 months under natalizumab treatment [147]. In contrast, recent findings at the New York Mount Sinai School of Medicine demonstrated that white matter microstructural deterioration still occurred in relapsing-remitting MS patients, classified as having NEDA over an approximate 2 year evaluation period [148].

### Body fluid markers

Although numerous contenders have been piloted over the past decades, only two body fluid markers are currently being used in routine MS practice: i.e. the presence of oligoclonal bands (OCBs) and an elevated IgG index, both in the cerebrospinal fluid (CSF). Both are helpful in establishing the diagnosis, and the former has showed potential as an independent predictor of conversion to MS in CIS and RIS subjects [149], but overall these measures do not show enough responsiveness to disease activity to be useful in clinical follow-up [150]. One of the most promising alternatives is neurofilament light chain (Nf-L). Neurofilaments are an abundant and cell-exclusive part of the cytoskeleton in neurons (where they play an important role in maintaining axonal calibre and functionality) which makes them excellent biomarker candidates of axonal damage [151]. Nf-L can be measured in CSF, and, following the development of highly sensitive single-molecule array assays [152], since recently also in serum, where concentrations are much lower than in the CSF [151]. Values in both compartments appear to be highly correlated to each other [153–155], but serum has the advantage of being much more easily accessible.

Neuro-axonal damage is the pathological substrate of disability in a large scale of neurological diseases. Elevated neurofilament concentrations have been found in the serum and CSF of patients with MS, but also in individuals with traumatic, vascular and other degenerative conditions of the CNS [151]. In MS, Nf-L levels in the CSF have been associated with clinical endpoints (e.g. degree of disability, disease activity and time since last relapse) for the first time in 1998 [156], findings that subsequently have been replicated and extended by several other and larger studies [151], even providing prognostic information over up to 15 years [157]. Baseline levels are significantly higher in patients with a CIS that will convert to MS [158], and predict disease activity over 2 years [159], but the predictive value for conversion is still considered weak at this time [158, 160]. At a group level, CSF concentrations appear to be responsive to treatment with natalizumab, fingolimod, mitoxantrone and rituximab in both relapsing and progressive MS. [151]

Returning to the crux question of this paper, we are not aware of studies demonstrating elevated body fluid neurofilament levels in patients with MS, as a result of neuronal homeostasis disruption and in the objectivated absence of brain atrophy. Nf-L values were found to be increased in the serum of subjects with a CIS [161], where less pronounced brain atrophy might be expected, but not in the CSF of those with a radiologically isolated equivalent [162]. Levels in both body fluids did correlate significantly with upcoming brain parenchymal fraction changes (coefficients for CSF were -0.564 over 3 years and -0.892 over 5 years) [158, 163, 164], but elevations at baseline are possibly be linked to a higher concurrent inflammatory activity [165], which may cause a pseudo-atrophy phenomenon in these subjects. On the other hand, with longer follow-up times, effects of pseudo-atrophy should decrease, and Petzold and co-workers reported a very high odds ratio (36, p < 0.01) for the development of pronounced BV loss after 15 years [166]. Some have reported a more modest predictive value [167–169], but, in general, these findings do support the idea that a neurofilament rise can precede the development of brain atrophy in MS.

## Conclusions

**Fig. 5:**
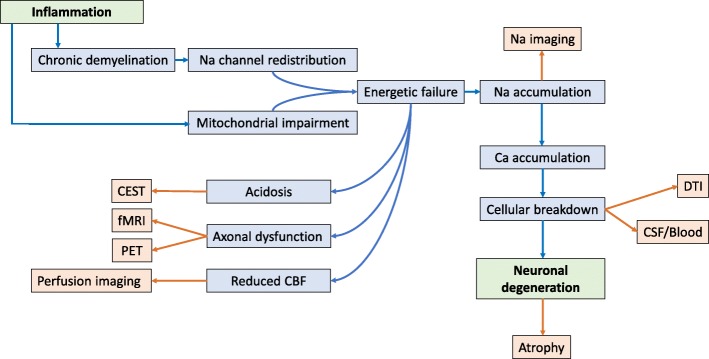
Biological background of potential early neurodegenerative markers in MS. Blue: biological processes possibly connecting inflammation with neurodegeneration in MS. Orange: techniques to address the potential corresponding biomarker. DTI: diffusion tensor imaging, CEST: chemical exchange saturation transfer, fMRI: functional magnetic resonance imaging, PET: positron emission tomography, CBF: cerebral blood flow, CSF: cerebrospinal fluid, MS: multiple sclerosis.

Despite the growing availability of efficacious disease-controlling therapy, MS remains a significant burden for global health. Accelerated BV loss has recently emerged as a popular imaging biomarker of neurodegeneration, which is considered to be the main determinant of long-term prognosis in affected individuals. Unfortunately, irreversible tissue damage is already likely at the time when brain atrophy can be reliably measured. This Review highlights potential neurodegenerative markers, that may precede these atrophic changes and, (Figure 5) therefore, possibly provide a more beneficial window of opportunity for treatment adjustment. One candidate is the detection of mitochondrial dysfunction, by means of decreased NAA levels, in normal-appearing white matter, which has been described as early as in the CIS and RIS stage. Reduced metabolic activity, all the more together with increased needs due to chronic demyelination and ion channel redistribution, may induce a chronic energy deficiency, resulting in intracellular sodium accumulation. Recent high-field MRI has demonstrated raised intracellular sodium concentrations in patients with relapsing-remitting MS, combined with decreased extracellular fractions, arguing against the concomitant presence of atrophy. Axonal metabolic dysfunction may also be associated with a reduced energy consumption and, hence, a reduced CBF, which has been demonstrated to be independent from atrophy. Yet, cerebral hypoperfusion may as well represent a distinct pathological mechanism in MS, caused by overexpression of the vasospastic agent endothelin-1. Next, microstructural fibral changes, as assessed by DTI and interpreted as reduced axonal integrity, were observed in the volumetrically normal hippocampus of subjects with a CIS, where they were associated with clinically relevant cognitive changes. Finally, Nf-L concentrations in blood and CSF have recently developed into a marker of axonal damage in a variety of neurological disorders. Elevated levels have been described in early stages of MS and appear to be predictive at the group level for the degree of future BV loss over a sizeable time period.

The statements and assumptions throughout this paper are not without limitations. First, definite proof for the proposed model linking inflammation to neurodegeneration in MS is, although plausible and frequently put forth in the literature, still lacking and therefore our basis remains hypothetical. MS is an extremely complex disease in which many other pathways may lead to neurodegeneration and atrophy, including primary deficits in neurons, oligodendrocytes or astrocytes [170–172]. But if this pathophysiological basis is correct, halting early inflammatory disease activity will prevent neurodegeneration and result in a better clinical outcome for newly diagnosed subjects. BV assessment is becoming increasingly incorporated in treatment trials for MS, with positive data for both licensed and more experimental agents [22, 173, 174]. Interestingly, it has recently been suggested that teriflunomide may reduce disability worsening mainly through its effects on BV preservation [175]. We hope that insights from our paper will help the development of biomarkers that enable treatment decisions to anticipate on brain atrophy/irreversible tissue damage, hereby further favoring clinical prognosis. Second, this paper was not written as a systematic review based on a general literature search. The authors provided input from their respective areas of expertise, grafted on the subcellular model for neurodegeneration in MS, and complemented by specific individual database revisions (e.g. for the section on cerebral perfusion, the items ‘brain volume’ and ‘brain atrophy’ were used in PubMed, in combination with ‘cerebral blood flow’ and ‘cerebral hypoperfusion’) and bibliographies of key publications. Third, it remains unsure whether therapeutic decisions based on advanced MRI or biochemical findings suggestive of axonal suffering eventually will ensure better clinical outcomes. Studies in this field are non-existing, individual cut-off values of the abnormal are unknown, and it cannot be excluded that subcellular damage reaches a point-of-no-return before we are able to detect it. Fourth, BV loss has been granted the central role as marker of the neurodegenerative process in MS, while other potential and emerging candidates such as spinal cord and retinal atrophy have not been addressed [176, 177]. Reduced retinal nerve layer thickness (RNFL), as measured with optical coherence tomography, has been associated with both measures of brain atrophy and inflammatory disease activity in subjects with MS. [178, 179] Pisa and colleagues have recently demonstrated that the RNFL was relatively preserved over approximately 2 years in NEDA patients with a relapsing-remitting subtype while this was not the case in those with a progressive clinical subtype but which were stable according to their EDSS score. Based on these findings, the authors suggested that the anterior optic pathways share the same mechanisms of degeneration as the rest of the CNS, rather than predominantly resulting from transsynaptic degeneration secondary to lesional activity in the optic radiation [180]. Retinal degeneration seems to be an interesting reflection of analogue brain processes but it remains unsure whether detecting the former may eventually serve as a prior warning for the latter, as a correlating coexistence of both has already been demonstrated very early in the disease [181–183]. Preliminary data suggest that mitochondrial dysfunction might by reflected by alternative imaging and body fluid biomarkers (e.g. uric acid) as well [184, 185]. Fifth, the techniques described in this Review are not free from reliability and reproducibility issues. A very specific technical expertise is usually required and the impact of several potential biological confounders (e.g. normal aging, exercise, hydration status) on all presented measures still needs to be fully elucidated. Advanced acquisition schemes are more difficult to reproduce across different scanner vendors and clinical sites, which can be illustrated by a recent meta-analysis indicating a poor intraclass correlation coefficient (0.29) of fMRI connectivity estimates [186]. Blood and CSF neurofilament levels can increase with growing age [187, 188], and research has been mainly focusing on (younger) relapsing-remitting patients while there is a relative lack of data in more advanced disease stages. Sixth, it has been widely acknowledged that depression, vascular and autoimmune comorbidities are more prevalent in patients with MS than in age-matched controls [5]. These comorbid conditions may affect clinical outcomes and brain pathology, and confound clinical research. Zivadinov and colleagues assessed a large cohort of 815 subjects with MS, of which 29.6% had autoimmune comorbidities and demonstrated a reduction of whole-brain and cortical volumes. The main autoimmune diseases affecting brain volumes were psoriasis, thyroid disease, and type 1 diabetes mellitus [189]. Additionally, Lorefice and co-workers observed a reduced grey matter volume in individuals with type I diabetes, which correlated with diabetes disease duration [190]. Our selection of candidate biomarkers is informed by the different pathological processes and does not take comorbidities into account. Also, the potential coexistence of other neurodegenerative disorders such as Alzheimer’s disease needs to considered in an ageing population [191]. As illustrated by the findings from the Nun and Honolulu-Asia Aging studies, the total burden of comorbid neuropathology may actually be more relevant for the development of clinical symptoms than any single lesion [192]. In this context, combining several biomarkers that assess different pathways resulting in neurodegeneration could be valuable.

Studies exploring BV in combination with metabolic or molecular MRI, perfusion assessment, connectivity analysis and/or neurofilament measurement are scarce but should guide new work in this field. Thus far, only CBF and DTI alterations have been directly evaluated against brain volumetry, and provide the highest level of evidence. In order to disentangle the different possible mechanisms leading to atrophy and their timelines, longitudinal and multimodal studies are required. Studies of particular interest would be to explore the longitudinal relationship between glutamate, NAA and BV, to investigate the hypothesized U-shaped curve of functional MRI connectivity, and to investigate whether metabolic dysfunction induces a reduced cerebral blood flow or the other way around. These studies could provide important novel insights in disease mechanisms and provide new pathways to counteract the neurodegenerative effects of MS before irreparable damage has occurred. Finally, it is worth noting that with the advent of big medical data and artificial intelligence (AI) techniques, new models can be generated that may be able to predict atrophy before irreversible tissue loss has set in. Yet, AI requires a huge amount of data that is currently unavailable for most of the modalities presented in this paper. A specific (imaging) modality should first have some clinical value before large datasets can be collected. Based on the current literature, we only found one study that applied a deep learning strategy to uncover sustained EDSS progression [193], that reached a modest area under the receiver operator characteristic curve of 0.66. Other papers have explored the possibility of using non-linear models (like support vector machines and artificial neural networks) with standard brain volumetric measurements as input [194, 195]. Although these models may help to predict clinical deterioration (EDSS score change), they are already based on atrophy estimates and thus fall beyond the scope of this paper.

## Data Availability

Not applicable.

## Abbreviations

AHSCT: Autologous stem cell transplantation
AI: Artificial intelligence
APT: Amide proton transfer
ASIC: Acid-sensing ion channel
ASL: Arterial spin labelling
ATP: Adenosine triphosphate
BBB: Blood-brain barrier
BOLD: Blood oxygenation level dependent
BV: Brain volume
CBF: Cerebral blood flow
CEST: Chemical exchange saturation transfer
CIS: Clinically isolated syndrome
CNS: Central nervous system
CSF: Cerebrospinal fluid
DMF: Dimethylfumarate
DTI: Diffusion tensor imaging
EDSS: Expanded Disability Status Scale
EMA: European Medical Agency
FA: Fractional anisotropy
FC: Functional connectivity
FDA: Food and Drug Administration
FDG: Fluorodeoxyglucose
fMRI: Functional magnetic resonance imaging
GABA: Gamma-aminobutyric acid
INF: Interferon
MD: Mean diffusivity
MRI: Magnetic resonance imaging
MRS: Magnetic resonance spectroscopy
MS: Multiple sclerosis
NAA: N-acetyl-aspartate
NEDA: No evidence of disease activity
Nf-L: Neurofilament light chain
NO: Nitric oxide
OCBs: Oligoclonal bands
PET: Positron emission tomography
RIS: Radiologically isolated syndrome
RNFL: Retinal nerve layer thickness
ROS: Reactive oxygen species
RR: Relapsing-remitting
RS-FC: Resting-state functional connectivity
