# Correction to: Detecting neurodegenerative pathology in multiple sclerosis before irreversible brain tissue loss sets in

**DOI:** 10.1186/s40035-019-0182-8

**Published:** 2020-01-03

**Authors:** Jeroen Van Schependom, Kaat Guldolf, Marie Béatrice D’hooghe, Guy Nagels, Miguel D’haeseleer

**Affiliations:** 1 Neurology Department, Universitair Ziekenhuis Brussel; Center for Neurosciences, Vrije Universiteit Brussel, Laarbeeklaan 101, 1090 Brussel, Belgium; 2 Radiology Department Universitair Ziekenhuis Brussel, Brussels, Belgium; 3 Nationaal Multiple Sclerose Centrum, Melsbroek, Belgium

**Correction to: Transl Neurodegener**

**https://doi.org/10.1186/s40035-019-0178-4**

In the original publication of this article [1], the following statement should be added in the Acknowledgement section:

This paper has been published with the support of the Universitaire Stichting van België.
